# Evaluation of antinociceptive activity of hydromethanol extract of *Cyperus rotundus* in mice

**DOI:** 10.1186/1472-6882-14-83

**Published:** 2014-03-04

**Authors:** Mohammad Zafar Imam, Chandra Datta Sumi

**Affiliations:** 1 Department of Pharmacy, Stamford University Bangladesh, 51 Siddeswari Road, Dhaka 1217, Bangladesh

**Keywords:** Analgesic, Cyperaceae, *Cyperus rotundus*, Medicinal plant, Pain

## Abstract

**Background:**

*Cyperus rotundus* Linn. (Cyperaceae) is used to treat inflammation, pain, fever, wounds, boils and blisters in folk medicine. This study evaluated the antinociceptive effect of the hydromethanol extract of whole plant of *C. rotundus* (HMCR).

**Methods:**

The antinociceptive activity of HMCR was investigated in thermal-induced (hot plate and tail immersion) and chemical-induced (formalin) nociception models in mice at three different doses (50, 100 and 200 mg/kg; p.o.). Morphine sulphate (5 mg/kg, i.p.) and diclofenac sodium (10 mg/kg, i.p.) were used as reference analgesic agents.

**Results:**

In the hot-plate and tail-immersion tests HMCR significantly increased the latency period to the thermal stimuli at all the tested doses (50, 100 and 200 mg/kg) (*p* < 0.05). The significant increase in latency is clear from the observations at 60 and 90 min. In formalin-induced paw licking test oral administration of HMCR at 100 and 200 mg/kg doses decreased the licking of paw in early phase. All the tested doses (50, 100 and 200 mg/kg) significantly decreased the licking of paw in late phase of the test (*p* < 0.001). The dose 200 mg/kg was most effective showing maximum percentage of inhibition of licking in both early (61.60%) and late phase (87.41%).

**Conclusion:**

These results indicate the antinociceptive effect of *C. rotundus* and suggest that this effect is mediated by both peripheral and central mechanisms. These results support the traditional use of this plant in different painful conditions.

## Background

The genus *Cyperus* includes common weeds found mainly in upland, paddy fields as well as marshy places in tropical, subtropical and temperate regions. *Cyperus rotundus* Linn., commonly known as nutgrass, is a perennial, herbaceous sedge with scaly creeping rhizomes and bulbous at the base. It is locally known as “Mutha”. The plant is a widely used traditional medicinal herb in India, China, Japan, Korea, Combodia, Nigeria, and Bangladesh. Mainly the rhizomatous tubers are used in stomach and bowel disorders, inflammatory diseases [1,2], as an analgesic, a sedative drug [1] etc. Besides many other uses, this plant is used in different painful conditions such as inflammation, pain, fever, wounds, boils and blisters [3]. Different chemical compounds such as alkaloids, flavonoids, tannins, starch, glycosides, furochromones, monoterpenes, sesquiterpenes, sitosterol, essential oil, fatty oil containing a neutral waxy substance, glycerol, linolenic, myristic and stearic acids and many other compounds have been isolated from the plant [3,4]. Pharmacological properties such as anti-candida [5], anti-inflammatory [6], antidiabetic [7], antidiarrhoeal [8,9], cytoprotective [10], antimutagenic [11], antimicrobial, antioxidant [12], antibacterial, cytotoxic and apoptotic [13,14], analgesic [15], anticonvulsant [16], and wound healing [17] activities have been reported.

The use of *C. rotundus* in different painful conditions in folk medicine but lack of scientific study reporting its antinociceptive activity in both chemical- and heat-induced nociception models convinced us to design the present study to evaluate the antinociceptive effect of hydromethanol extract of the whole plant of *C. rotundus*.

## Methods

### Plant materials and extract preparation

The whole plant of *C. rotundus* Linn. was collected from Manikgonj district of Bangladesh in October, 2012. The collected plants were then identified by the experts of National Herbarium, Mirpur, Dhaka, Bangladesh (Accession No. 37861) where a voucher specimen has been deposited for further reference. The whole plant samples were washed, dried, grounded and 250 g of the dried powder was taken in a beaker. Then methanol and water (70:30) was added at the amount of 840 ml and 360 ml respectively and it was kept for three days with occasional stirring. Then it was filtered using a sterilized cotton filter and dried using rotary evaporator. After drying, 21.9 g (yield 8.76%) of dried extract was obtained from 250 g of powder. This crude extract was used for the investigation.

### Chemicals

Diclofenac sodium (Square Pharmaceuticals Ltd., Bangladesh), Morphine sulphate (Gonoshasthaya Pharmaceuticals Ltd., Bangladesh), 0.9% Sodium chloride solution (Normal saline) (Orion Infusion Ltd., Bangladesh), Formalin (Merck, Germany) and other reagents were of analytical grade.

### Animals

Swiss Albino mice (20-25 g) were collected from the Animal Resources Branch of the International Center for Diarrhoeal Disease Research, Bangladesh (ICDDR,B). Animals were housed in cages and were maintained under standard environmental conditions (Temperature: 24.0 ± 1.0°C; relative humidity: 55-65%; 12 hrs light/12 hrs dark cycle). Pellets of mice food prepared by ICDDR,B were given to the mice with fresh water *ad libitum*. All the experimental animals were treated following the Ethical Principles and Guidelines for Scientific Experiments on Animals (1995) formulated by The Swiss Academy of Medical Sciences and the Swiss Academy of Sciences. The study protocol was approved by the Ethics Committee of Stamford University Bangladesh.

### Acute toxicity test

Mice were divided into control and four test groups (n = 5). The test groups received HMCR orally at the doses of 1000, 1500, 2000, and 3000 mg/kg body weight. After gavage the animals were kept in separate cages and were allowed to food and water *ad libitum*. The animals were then observed for possible behavioral changes, allergic reactions (skin rash, itching) and mortality for the next 72 h [18].

### Hot plate test

The hot-plate test was performed according to the method described by Eddy and Leimbach (1953) [19] with slight modification. The temperature of the metal surface of Eddy’s hotplate was set at 52 ± 2°C. The mice that showed fore paw licking, withdrawal of the paw(s) or jumping response within 15 s on hotplate were selected for this study 24 h prior to the experiment. Mice were fasted overnight with water given *ad libitum*. The mice were then treated with 0.9% sodium chloride solution as vehicle (0.1 ml/mice, p.o.), HMCR (50, 100, and 200 mg/kg), and morphine as positive control (5 mg/kg, i.p.). HMCR was administered (50, 100 and 200 mg/kg, p.o.) 30 min before the experiment while morphine sulphate was administered (5 mg/kg, i.p.) 15 min before the experiment. The response in the form of fore paw licking, withdrawal of the paw(s) or jumping was recorded at 30, 60, 90, and 120 min following treatment. A cut off period of 20 s was maintained to avoid paw tissue damage. The results of the hot plate test are expressed as a percentage of the maximal possible effect (%MPE), which was calculated using following formula:

$$\%\mathrm{MPE}=\left[\left(\mathrm{Postdrug}\mathrm{latency}-\mathrm{predrug}\;\mathrm{latency}\right)/\left(\mathrm{Cut}\;\mathrm{off}\;\mathrm{period}-\mathrm{predrug}\;\mathrm{latency}\right)\right]\times 100.$$

### Tail immersion test

This test is based on the observation that morphine like drugs selectively prolongs the reaction time of the typical tail-withdrawal reflex in mice [20,21]. HMCR was administered (50, 100 and 200 mg/kg, p.o.) 30 min before the experiment and morphine sulphate was administered (5 mg/kg, i.p.) 15 min before the experiment. 1 to 2 cm of the tail of mice was immersed in warm water kept constant at 52 ± 1°C. The reaction time was the time taken by the mice to deflect the tail. The latency period of the tail-withdrawal response was taken as the index of antinociception and was determined at 30, 60, 90, and 120 min after the treatments. To determine the baseline, each animal was tested before administration of drug/extract. The %MPE was calculated using the same formula used in hot plate test.

### Formalin-induced paw licking test

Formalin-induced paw licking test was performed as describe by Santos and Calixto [22] and Santos et al. [23]. 20 μL of 2.5% formalin solution (0.92% of formaldehyde), made up in saline water, was injected into the sub-plantar area of the right hind paw of mice. Animals were pre-treated with different doses of HMCR (50, 100 and 200 mg/kg, p.o.) 60 min before formalin injection. Control group received only the vehicle (0.1 ml/kg saline water). Mice treated with morphine (5 mg/kg, i.p.) 15 min before the formalin injection was categorized as positive control. Licking of the injected paw was counted from 0-5 min (first phase) and 15-30 min (second phase) after formalin injection, corresponding to the neurogenic and inflammatory pain responses, respectively. The initial response was initially attributed to a direct algogenic effect of formalin on the nociceptors whereas phase two was associated with the release of local endogenous mediators responsible for sensitization of primary and spinal sensory neurons and subsequent activation of the nociceptors [24].

### Statistical analysis

The results are presented as Mean ± SEM. The one-way ANOVA test with Dunnett’s post hoc test was used to analyze the data using SPSS 11.5 software. *p* < 0.05-0.001 were considered as statistically significant.

## Results and discussion

### Acute toxicity

Administration of HMCR at the doses 1000, 1500, 2000 and 3000 mg/kg did not cause any mortality, behavioral changes, or allergic reactions. So it can be said that LD_50_ of *C. rotundus* is more than 3000 mg/kg and therefore it showed low toxicity profile.

### Hot plate test

Table 1 shows the antinociceptive effect of HMCR and standard drug (Morphine) assessed using the hot plate test. HMCR, at the doses of 100 and 200 mg/kg, exhibited significant (*p <* 0*.*001) ability to prolong the latency of response to thermal-induced nociception throughout the whole experimental period. The effect was dose-dependent and HMCR showed stronger effect at 100 and 200 mg/kg doses. The extract showed significant %MPE at 50, 100 and 200 mg/kg doses.

**Table 1:** **Antinociceptive effect of *C. rotundus *** **extract and morphine in hot plate test**

**Treatment**	**Dose (mg/kg)**	**Response time (s) (%MPE)**				
		**Pretreatment**	**30 min**	**60 min**	**90 min**	**120 min**
Vehicle	0.1 ml/mice	7.44 ± 0.48	7.89 ± 0.62	7.52 ± 0.86	7.91 ± 0.90	8.44 ± 0.83
Morphine sulphate	5	8.70 ± 0.72	14.89 ± 1.01**(54.72)	18.17 ± 0.66**(83.76)	14.70 ± 0.26**(53.04)	12.99 ± 0.93(37.94)
HMCR	50	8.22 ± 0.91	10.27 ± 0.55(17.45)	11.97 ± 0.28*(31.89)	10.85 ± 0.90(22.35)	9.22 ± 1.32(8.52)
HMCR	100	6.19 ± 0.91	10.11 ± 0.72(28.38)	13.03 ± 1.18**(49.55)	11.63 ± 0.97*(39.43)	10.77 ± 0.83(33.17)
HMCR	200	8.22 ± 0.72	12.21 ± 0.62*(33.89)	13.93 ± 0.63**(48.51)	11.99 ± 0.76*(32.04)	11.07 ± 1.47(24.19)

Each value is presented as the mean ± SEM (n = 5); *p < 0.05 compared with the control group (Dunnett’s test). **p < 0.001 compared with the control group (Dunnett’s test).

The hot-plate test is a specific test carried out to verify involvement of central mechanism with compounds/drugs showing antinociceptive activity [25]. In this work, HMCR showed a marked inhibition on thermal‒induced hyperalgesia as it showed significant increase in latency (p < 0.001) compared to control. Morphine (5 mg/kg i.p.) was used as a standard drug which demonstrated a stronger analgesic effect than HMCR. The effect was evident from the elongation of the latency time till the 3^rd^ observation (90 min). The effect of compounds or plant extract in such mechanism by increasing the latency are suggested to act like centrally mediated drugs [26] by activating the periaqueductal gray matter (PAG) to release endogenous peptides (i.e., endorphin or enkephalin). These endogenous peptides descend the spinal cord and function as inhibitors of the pain impulse transmission at the synapse in the dorsal horn [27].

### Tail immersion test

The tail-withdrawal reflex time of the mice to the hot water-induced pain was also significant after administration of HMCR (Table 2). The effect of HMCR at 100 and 200 mg/kg doses at 60 and 90 min was significant (*p* < 0.05) in comparison to control while at dose 50 mg/kg it did not show any significant increase in latency. The maximum effect of the extract was recorded at 60 min. However, the increase in latency was less significant than that observed in the hot plate test. Morphine at 5 mg/kg showed highest %MPE values while the extract also showed significant %MPE at 100 and 200 mg/kg doses (*p* < 0.05) at different observation time.

**Table 2:** **Antinociceptive effect of *C. rotundus *** **extract and morphine in tail immersion test**

**Treatment**	**Dose (mg/kg)**	**Response time (s) (%MPE)**				
		**Pretreatment**	**30 min**	**60 min**	**90 min**	**120 min**
Vehicle	0.1 ml/mice	2.83 ± 0.48	2.62 ± 0.37	2.68 ± 0.46	2.66 ± 0.43	2.59 ± 0.48
Morphine sulphate	5	2.66 ± 0.56	5.42 ± 0.47**(37.62)	6.21 ± 0.41**(48.32)	4.64 ± 0.31*(26.00)	3.57 ± 0.40(12.45)
HMCR	50	2.31 ± 0.37	3.25 ± 0.47(12.22)	3.98 ± 0.17(21.71)	3.14 ± 0.12(10.87)	2.62 ± 0.24(4.03)
HMCR	100	2.66 ± 0.12	3.66 ± 0.35(13.61)	4.52 ± 0.32*(25.33)	3.88 ± 0.34*(16.61)	2.97 ± 0.17(4.30)
HMCR	200	2.54 ± 0.49	3.87 ± 0.35(17.88)	4.44 ± 0.36*(25.47)	4.16 ± 0.34*(21.66)	3.14 ± 0.24(8.04)

Each value is presented as the mean ± SEM (n = 5); *p < 0.05 compared with the control group (Dunnett’s test), **p < 0.001 compared with the control group (Dunnett’s test).

Tail immersion model is considered as an acute pain model. The tail-withdrawal response of mice is predominantly considered to be selective for centrally acting analgesics, whereas the peripherally acting drugs are known to be inactive on such heat-induced pain response [28]. The significant increase (*p* < 0.05) in tail-withdrawal time by the extract suggests the involvement of central mechanisms in its antinociceptive effect. Both tail immersion and hot plate test measure the latency time of mice to thermal stimuli. Tail immersion monitors a spinal reflex involving μ_2_- and δ-opioid receptors, whereas the hot plate demonstrates supraspinal reflex mediated by μ_1_- and μ_2_-opioid receptors [29]. Therefore, the results of the present study indicate that the central antinociceptive effect of *C. rotundus* may be prominent on μ-opioid receptors.

### Formalin-induced paw licking test

HMCR has shown a dose-dependent antinociceptive effect in both phases of Formalin test. HMCR (50, 100 and 200 mg/kg p.o.) significantly (*p* < 0.001) reduced the number of paw licking in both phases of the test when compared to control group. Morphine and diclofenac sodium, used as positive control decreased the licking significantly compared to control in both phases (Table 3).

**Table 3:** **Antinociceptive effect of *C. rotundus *** **extract, morphine and diclofenac sodium in formalin-induced paw licking test**

**Treatment**	**Dose (mg/kg)**	**Number of licking**			
		**Early phase (0-5 min)**	**% inhibition**	**Late phase (15-30 min)**	**% inhibition**
Vehicle	0.1 ml/mice	157.00 ± 8.21	-	189.00 ± 6.42	-
Morphine sulphate	5	44.20 ± 5.44*	71.99	8.40 ± 1.81*	95.56
Diclofenac sodium	10	89.60 ± 7.41*	43.22	35.40 ± 2.86*	81.27
HMCR	50	135.20 ± 7.12	14.32	90.80 ± 8.64*	51.96
HMCR	100	89.40 ± 6.88*	43.35	51.40 ± 9.47*	72.80
HMCR	200	60.60 ± 9.32*	61.60	23.80 ± 5.08*	87.41

Each value is presented as the mean ± SEM (n = 5); *p < 0.001 compared with the control group (Dunnett’s test).

In formalin-induced paw licking test HMCR has shown the ability to affect both the early and late phase inflammatory effects of the formalin test, which implies the involvement of not only the central mechanism but also the peripheral antinociceptive activity of the extract. The early phase, classified as neurogenic pain, is an acute response observed immediately after the administration of formalin and is due to direct action of injected formalin on nociceptors. While the late phase, classified as an inflammatory pain, is a late response resulting from the inflammatory processes generated by the release of inflammatory mediators such as histamine, serotonin, prostaglandins and bradykinin, and activation of the neurons in the dorsal horns of the spinal cord [30]. Both phases have their own characteristics that can be used as tool to assess the antinociceptive potential as well as to elucidate the mechanisms of antinociception. The early phase represents a direct irritant effect of formalin on sensory fibers, while the late phase represents response secondary to the development of inflammatory process and the release of inflammatory mediators [31]. It has been reported that drugs acting centrally (i.e. narcotics/opioids) inhibit both phases of the formalin test while those acting peripherally (i.e. NSAIDs) inhibit only the late phase, respectively [32,33]. Therefore, the results shown by HMCR suggest that the extract contains bioactive compound(s) with central and peripheral antinociceptive actions and additional anti-inflammatory activity [30]. The ability of HMCR to inhibit chemically- and thermally-induced nociceptive processes tested in this study presents its potential to be used as an analgesic agent.

## Conclusion

Results of the present study indicate that all tested doses of HMCR exhibited significant central and peripheral antinociceptive effect. The effect is rapid, long lasting, and statistically significant particularly at 100 and 200 mg/kg doses. Taking these findings into account, it seems quite possible that *C. rotundus* contains constituents with promising antinociceptive activity. The traditional use of the plant in the treatment of painful conditions can be affirmed by this study. However, further studies are required to isolate the bioactive compounds and elucidate the precise mechanisms responsible for the antinociceptive activity.

## Competing interests

The authors declare that they have no competing interests.

## Authors’ contributions

MZI conceived, designed and coordinated the study. CDS conducted the study. MZI and CDS performed the statistical analysis, interpreted the data and drafted the manuscript. Both authors read and approved the final manuscript.

## Pre-publication history

The pre-publication history for this paper can be accessed here:

http://www.biomedcentral.com/1472-6882/14/83/prepub
